# Adapting to Constraints: Successful Repair of a Challenging Tetralogy of Fallot Case

**DOI:** 10.1155/crpe/4690543

**Published:** 2026-08-24

**Authors:** Oung Savly, Sujith S. Pereira, H. Y. Soklay

**Affiliations:** ^1^ Paediatric Cardiology, Kantha Bopha Children’s Hospital, Phnom Penh, Cambodia; ^2^ Neonatal Unit, Homerton University Hospital, Homerton Healthcare NHS Foundation Trust, London, UK, nhs.uk; ^3^ Child Health, Blizard Institute, Barts and the London School of Medicine and Dentistry, London, UK, qmul.ac.uk; ^4^ Paediatric Cardiac Surgery, Kantha Bopha Children’s Hospital, Phnom Penh, Cambodia

**Keywords:** challenges, surgical repair, Tetralogy of Fallot

## Abstract

This case describes the clinical presentation and management of a 5‐month‐old infant who presented with cyanosis worsened with crying and failure to thrive. He was diagnosed with Tetralogy of Fallot using detailed echocardiography, which was used to plan corrective surgery and achieve the best outcome for this infant, who was successfully discharged home 1 week following surgery. This case report highlights how these challenging cases can be managed as per international recommendations and provides an insight into the practical aspects of caring for such challenging cases in a resource‐limited setting.

## 1. Introduction

The evolution in cardiology and cardiothoracic surgery, since Steno first described this defect in 1673 and subsequently Fallot of Marseilles who first coined the term in 1888, has been remarkable for Tetralogy of Fallot (ToF), the most common cyanotic congenital heart disease [1]. Complete surgical repair with or without previous Blalock–Taussig shunt palliation was the standard of care but with further advances including minimally invasive procedures such as ductal shunting, these infants can be palliated until an age where corrective repair is associated with less morbidity and mortality [2]. Surgical repair has evolved over the years with refinements in surgical techniques including percutaneous and hybrid interventions for postoperative management [3]. Paediatric cardiac care provision can vary between resource limited and resource plentiful settings [4], but there remains a paucity on the management of such case in resource‐limited settings. This case report aims to describe the practical challenges faced during care provision for this challenging case in a resource limited setting.

## 2. Case Presentation

**FIGURE 1:**
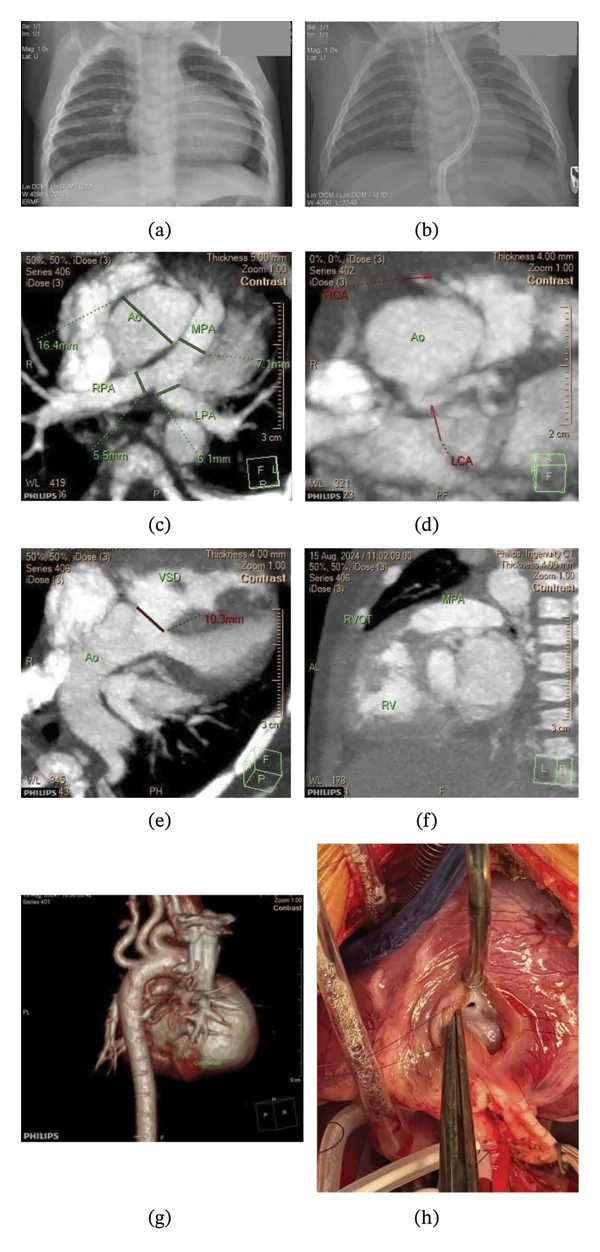
Preoperative chest X‐ray (a) showing the typical boot‐shaped heart with relative oligaemic lung fields when compared to postoperative chest X‐ray (b). Good size branch pulmonary arteries (c) and normal origin of the coronaries (d). Large ventricular septal defect (e) with severe infundibular stenosis (f). There was evidence of a few collaterals (g) and severe infundibular stenosis intra‐op (h).

A 5‐month‐old infant presented to our hospital with bluish discolouration of lips made worse during crying since 1 month of age. He weighed 5 kg and had a systolic murmur with oxygen saturations of 65% in room air. His chest X‐ray revealed a boot‐shaped heart (Figure 1a). Multimodality investigations demonstrated a 10‐mm ventricular septal defect (VSD) (Figure 1e) with right to left flow, overriding aorta, bicuspid pulmonary valve measuring 8 mm (z −1.68) with infundibular stenosis (Figure 1f) with a pressure gradient of 80 mmHg and right ventricular hypertrophy; findings consistent with ToF. His left‐sided aortic arch showed no signs of coarctation or patent ductus arteriosus. However, he had a few small collaterals (Figure 1g). The coronary arteries and pulmonary venous return were normal. During admission, he developed Tet spells on multiple occasions, which were managed with opioids and volume. He continued to have severe Tet spells, requiring further sedation and fluid resuscitation. Following the multidisciplinary team meeting, the team repaired his defect considering his age and difficult clinical course. Intraoperatively, critical infundibular stenosis was found (Figure 1h). His VSD closure and infundibular resection were uneventful. He was extubated on post‐op Day 1, and inotropic support was weaned off by post‐op Day 2. He was discharged home by post‐op Day 7 with follow‐up.

## 3. Discussion

ToF is associated with a syndromic diagnosis in 20% of cases [5]. Hypercyanotic or Tet spells result from dynamic obstruction of the right ventricular outflow tract due to narrowing of the right ventricular infundibulum during ventricular contraction. This causes right‐to‐left shunting via the VSD and reduced pulmonary blood flow. As the right ventricular muscle hypertrophies with time, the risk of these Tet spells increases over time. The ideal timing of surgery is between 3 and 11 months, but symptomatic neonates may require earlier intervention [6]. Corrective repair performed in the neonatal period has been associated with increased mortality, longer intensive care days and hospital stay [7–9].

Staged repair with shunt palliation and subsequent complete repair when compared with primary repair in a cohort of infants under 4 months showed similar cardiac‐related mortality and reintervention at a median follow‐up of 8 years [10]. In another cohort of 6021 infants, where 2030 (34%) infants who underwent intervention for ToF, the in‐hospital mortality between primary repair (6%), shunt (6%) and ductal stenting (3%) did not differ. Pulmonary annular z scores of less than or equal to −3 is seen in severe cases making corrective repair challenging [11]. A large cohort of infants showed that a postoperative right ventricular outflow tract peak gradient of less than 25 mmHg prevented reoperation [12]. There has been a reduction in mortality over the decades following surgery from 0.52 in the 1979s to 0.19 in 2010s but when compared with matched well controls, the mortality remains significantly high [13].

At our centre, complete repair of ToF is generally performed after 6 months of age. In practice, surgery is usually undertaken earlier when oxygen saturation falls below 85%, or when recurrent hypercyanotic spells occur as described in the index case.

In smaller infants, primary repair can be technically more challenging because of fragile myocardial tissue and smaller intracardiac structures necessitating the skills of an experienced heart surgeon. Additionally, younger infants who require transannular patch enlargement may face a higher likelihood of significant postoperative pulmonary regurgitation (PR). In our setting, smaller Contegra grafts are not always suitable or available for this age and weight group; however, pulmonary valve reconstruction can be performed using the right atrial appendage as our alternative strategy. Accordingly, the long‐term burden of free PR must be carefully considered when planning early repair. Therefore, in clinically stable patients, delaying surgery until later infancy is often considered to provide a more favourable balance between surgical complexity and postoperative outcome.

In our case, preoperative echocardiography demonstrated favourable pulmonary valve annulus, suggesting a possibility of valve‐sparing repair. This was a key determinant in management, as pulmonary valve preservation could reduce the risk of severe postoperative PR. For these reasons, early complete repair was considered preferable to postponement or staged palliation BTT shunt, avoiding shunt‐related complications, and later reoperation.

This case also highlights the pivotal role of expert transthoracic echocardiography in a resource‐constrained setting. Comprehensive echocardiographic assessment provided sufficient anatomical and physiological information regarding RVOT obstruction, pulmonary annulus size, branch pulmonary arteries, coronary arteries and ventricular function to support definitive surgical planning. Advanced imaging, such as cardiac MRI or CT, is not routinely necessary. High‐quality echocardiography remains the primary imaging modality for many infants with straightforward ToF anatomy.

Our hospital is the largest paediatric cardiac referral centre in Cambodia and receives a high volume of congenital heart disease cases nationwide. Centralisation of services results in improved expertise from exposure to high volume of pathology both for the cardiologist and the cardiac surgeons. Centralisation of specialised healthcare service provision has been shown to be associated with improved outcomes, lower length of stay, improved quality of care and life and economic benefits [14]. Additionally, free service provision does not deter referral of cases of varied complexities and challenges. Free service provision has been shown to be associated with improved attendance in both outpatient and inpatient care [15, 16].

Timely definitive repair in resource‐limited environments depends not only on anatomy and physiology but also on the availability of surgical expertise, blood bank support, intensive care capacity and equitable healthcare access. Cardiopulmonary bypass is routinely performed across a wide range of ages and body weights which is made possible due to a dedicated, well equipped and staffed cardiac intensive care unit.

In smaller infants with severe pulmonary stenosis, postoperative pulmonary overcirculation and lung oedema can be challenging; therefore, we generally favour extubation after postoperative Day 2. Inotropes are weaned when haemodynamically appropriate.

We also run a dedicated training program for junior paediatric cardiologists in echocardiography and paediatric cardiac intensive care and collaborate with paediatricians nationwide to improve referral pathways. As a result, referrals have increased progressively, with fewer children presenting late. This model of training has potential for further growth with the help of telemedicine [17, 18].

This case illustrates that in selected symptomatic infants with favourable anatomy, early complete valve‐sparing repair can be safely achieved in a limited‐resource setting when supported by experienced teams.

## Author Contributions

Oung Savly collected the clinical data, acquired echocardiographic images and contributed to the final version of the manuscript. Sujith S. Pereira conceptualised the study. Sujith S. Pereira drafted the manuscript and revised the manuscript based on feedback. H. Y. Soklay operated on the case and contributed to the final version of the manuscript.

## Funding

The authors have nothing to report.

## Disclosure

All authors have approved the final version of the study protocol.

## Ethics Statement

Ethical approval was waived by the Kantha Bopha Children’s Hospital, Phnom Penh, Cambodia, as this is a single anonymised case report that does not compromise patient confidentiality.

Written informed consent was obtained from the parent of the patient for publication of this case report.

## Consent

Please see the Ethics Statement.

## Conflicts of Interest

The authors declare no conflicts of interest.

## Supporting Information

Additional supporting information can be found online in the Supporting Information section.

## Supporting information


**Supporting Information 1** File 1: Preoperative echocardiogram showing a small pulmonary valve with turbulent flow across it when compared with the aortic valve.

**Supporting Information 2** File 2: Postoperative echocardiogram following pulmonary infundibular resection showing improved blood flow across the pulmonary valve.

**Supporting Information 3** CARE Checklist of information to include when writing a case report.

## Data Availability

Data used to support the findings of this study are available from the corresponding author upon reasonable request.
