# Supplementary material for: Adapting to Constraints: Successful Repair of a Challenging Tetralogy of Fallot Case
Source: Case Rep Pediatr. 2026 Aug 24;2026:4690543. doi: 10.1155/crpe/4690543 (PMC13501896; doi:10.1155/crpe/4690543)
Supplement: Supplementary file 1 [file CRPE-2026-4690543-s003.pdf]

CARE case report guidelines

# CARE Checklist of information to include when writing a case report

(cc) BY-NC-ND

| Topic | Item | Checklist item description | Reported on Line |
|---|---|---|---|
| **Title** | 1 | The diagnosis or intervention of primary focus followed by the words "case report" | 1 |
| **Key Words** | 2 | 2 to 5 key words that identify diagnoses or interventions in this case report, including "case report" | 1 |
| **Abstract (no references)** | 3a | Introduction: What is unique about this case and what does it add to the scientific literature? | 3 |
| | 3b | Main symptoms and/or important clinical findings | 3 |
| | 3c | The main diagnoses, therapeutic interventions, and outcomes | 3 |
| | 3d | Conclusion—What is the main "take-away" lesson(s) from this case? | 3 |
| **Introduction** | 4 | One or two paragraphs summarizing why this case is unique (**may include references**) | 4 |
| **Patient Information** | 5a | De-identified patient specific information. | 4 |
| | 5b | Primary concerns and symptoms of the patient. | 4 |
| | 5c | Medical, family, and psycho-social history including relevant genetic information | 4 |
| | 5d | Relevant past interventions with outcomes | 4 |
| **Clinical Findings** | 6 | Describe significant physical examination (PE) and important clinical findings. | 4 |
| **Timeline** | 7 | Historical and current information from this episode of care organized as a timeline | 4 |
| **Diagnostic Assessment** | 8a | Diagnostic testing (such as PE, laboratory testing, imaging, surveys). | 4 |
| | 8b | Diagnostic challenges (such as access to testing, financial, or cultural) | 4 |
| | 8c | Diagnosis (including other diagnoses considered) | 4 |
| | 8d | Prognosis (such as staging in oncology) where applicable | 5 |
| **Therapeutic Intervention** | 9a | Types of therapeutic intervention (such as pharmacologic, surgical, preventive, self-care) | 5 |
| | 9b | Administration of therapeutic intervention (such as dosage, strength, duration) | 5 |
| | 9c | Changes in therapeutic intervention (with rationale) | 5 |
| **Follow-up and Outcomes** | 10a | Clinician and patient-assessed outcomes (if available) | 5 |
| | 10b | Important follow-up diagnostic and other test results | 5 |
| | 10c | Intervention adherence and tolerability (How was this assessed?) | 5 |
| | 10d | Adverse and unanticipated events | 5 |
| **Discussion** | 11a | A scientific discussion of the strengths AND limitations associated with this case report | 5-8 |
| | 11b | Discussion of the relevant medical literature **with references**. | 5-8 |
| | 11c | The scientific rationale for any conclusions (including assessment of possible causes) | 5-8 |
| | 11d | The primary "take-away" lessons of this case report (without references) in a one paragraph conclusion | 5-8 |
| **Patient Perspective** | 12 | The patient should share their perspective in one to two paragraphs on the treatment(s) they received | 5-8 |
| **Informed Consent** | 13 | Did the patient give informed consent? Please provide if requested Yes | ~~Yes~~ ☐ ~~No~~ ☐ |
